# Niacin Skin Sensitivity Is Increased in Adolescents at Ultra-High Risk for Psychosis

**DOI:** 10.1371/journal.pone.0148429

**Published:** 2016-02-19

**Authors:** Gregor E. Berger, Stefan Smesny, Miriam R. Schäfer, Berko Milleit, Kerstin Langbein, Uta-Christina Hipler, Christine Milleit, Claudia M. Klier, Monika Schlögelhofer, Magdalena Holub, Ingrid Holzer, Michael Berk, Patrick D. McGorry, Heinrich Sauer, G. Paul Amminger

**Affiliations:** 1 University Hospital of Child and Adolescent Psychiatry, University of Zurich, Neumünsterallee 9, 8032 Zurich, Switzerland; 2 Department of Psychiatry, Jena University Hospital, Philosophenweg 3, D-07743 Jena, Germany; 3 Orygen Youth Health Research Centre, The University of Melbourne, Locked Bag 10, 35 Poplar Road Parkville, Victoria 3052, Melbourne, Australia; 4 Department of Child and Adolescent Psychiatry, Medical University of Vienna, Währingergürtel 18–20, A–1090 Vienna, Austria; 5 Department of Dermatology, University Hospital Jena, Erfurter Straße 35, D-07743 Jena, Germany; 6 Department of Nutritional Sciences, University of Vienna, Althanstrasse 14, A-1090 Vienna, Austria; 7 Deakin University of Melbourne, School of Medicine, Barwon Health, Geelong, Australia; 8 Florey Institute for Neuroscience and Mental Health, Parkville, Australia; Central Institute of Mental Health, Heidelberg University, GERMANY

## Abstract

**Background:**

Most studies provide evidence that the skin flush response to nicotinic acid (niacin) stimulation is impaired in schizophrenia. However, only little is known about niacin sensitivity in the ultra-high risk (UHR) phase of psychotic disorders.

**Methods:**

We compared visual ratings of niacin sensitivity between adolescents at UHR for psychosis according to the one year transition outcome (UHR-T n = 11; UHR-NT n = 55) with healthy controls (HC n = 25) and first episode schizophrenia patients (FEP n = 25) treated with atypical antipsychotics.

**Results:**

Contrary to our hypothesis niacin sensitivity of the entire UHR group was not attenuated, but significantly increased compared to the HC group, whereas no difference could be found between the UHR-T and UHR-NT groups. As expected, niacin sensitivity of FEP was attenuated compared to HC group. In UHR individuals niacin sensitivity was inversely correlated with omega-6 and -9 fatty acids (FA), but positively correlated with phospholipase A_2_ (*in*PLA_2_) activity, a marker of membrane lipid repair/remodelling.

**Conclusions:**

Increased niacin sensitivity in UHR states likely indicates an impaired balance of eicosanoids and omega-6/-9 FA at a membrane level. Our findings suggest that the emergence of psychosis is associated with an increased mobilisation of eicosanoids prior to the transition to psychosis possibly reflecting a “pro-inflammatory state”, whereas thereafter eicosanoid mobilisation seems to be attenuated. Potential treatment implications for the UHR state should be further investigated.

## Introduction

The observation that human kind responds with a red flush reaction of the upper body after intake of large doses of nicotinic acid (niacin, vitamin B3) goes back to the early fifties of the last century [1] a time when nicotinic acid was introduced as possible treatment of schizophrenia [2] While controlled studies failed to demonstrate a sustained clinical benefit of niacin in schizophrenia treatment [3] the clinical observation of an attenuated or even absent niacin flush reaction of the upper body in patients with schizophrenia compared to healthy controls caught the attention of some researchers [4,5]. The latter observation combined with other clinical observations such as that patients with schizophrenia are less sensitive to pain [6], have a lower temperature sensitivity [7] and a markedly decreased risk of rheumatoid arthritis [8], led to the proposal that schizophrenia might be associated with a deficiency or dysfunction of prostaglandin metabolism [9]. Prostaglandins are metabolites of arachidonic acid (AA), a key modulator of signal transduction [10]. The latter in conjunction with the observation that in particular AA levels are reduced in *post mortem* and *peripheral* red blood cell membranes in schizophrenia [11] lead to the formulation of the membrane and phospholipid hypothesis of schizophrenia [12,13].

The pathway underlying the niacin skin reaction includes the activation of G protein-coupled nicotinic acid receptors on skin macrophages and epidermal Langerhans cells that release AA from membrane phospholipids. As a next step a sequential oxidation of AA, and (to smaller quantities) also of dihomogamma-linolenic (DGLA) or eicosapentaenoic acid (EPA) by cyclooxygenases (COX-1 and COX-2) follows. Finally, prostaglandin synthases are responsible for the formation of a range of prostaglandins (e.g. D2, E2) that stimulate the production of cyclic AMP, which in turn triggers capillary vasodilatation and increase regional blood flow, observable as regional skin flushing [14,15].

A recent systematic review identified over 30 niacin sensitivity studies in schizophrenia and related disorders [16]. The majority of studies using the topical variant of the niacin skin test [17] reported that 23% up to 90% of patients with schizophrenia have an attenuated or absent flush reaction compared to less than 0–25% of the normal population [17–22]. Niacin sensitivity seemed to be more attenuated in first-episode than in multi-episode schizophrenia patients [23], raising the question if altered niacin sensitivity, as well as altered PUFAs and eicosanoid metabolism may be more relevant for the onset of psychosis than for chronic illness stages.

The identification of young people with a high risk for schizophrenia and other psychotic disorders has become a worldwide focus of attention [24]. Different clinical approaches have been applied to identify young people with an at risk mental state for psychosis, such as the Melbourne ultra-high risk (UHR) criteria [25–27] and the basic symptom concept [28,29]. To our knowledge at the current state of research only clinically defined approaches found their way into clinical daily routine and are able to identify help-seeking young people with an about 20% risk (with a range between 10–50%) to progress to a full-blown psychotic disorder within one year [30]. Most clinicians would agree that clinically defined help-seeking UHR individuals already need some kind of treatment, or at least intensive monitoring. However, there is also a clear need to further optimize the so far available UHR criteria. Following this demand, a range of biological markers, such as structural [31–33] and functional [34] imaging, neuropsychological [35], metacognitive [26,36,37] and electrophysiological [38] risk markers have been proposed. To our knowledge, alterations of niacin sensitivity in UHR populations have never been systematically investigated in this context. The few niacin sensitivity studies in first-degree relatives of patients with schizophrenia (with no drop in functioning in contrast to the UHR genetic liability subgroup) are inconclusive [14,39].

Therefore, our first aim in this study was to investigate niacin sensitivity in adolescents meeting the Melbourne UHR criteria compared to first episode psychosis patients (FEP), who meet criteria for schizophrenia or schizophreniform disorder, as well as to healthy controls (HC). Our second aim was to investigate if baseline niacin sensitivity differed between those who progressed towards psychosis (converters) versus those who did not (non-converters). Our third aim was to address the relationship of niacin sensitivity with other markers of phospholipid and fatty acid (FA) metabolism established in schizophrenia research, such as intracellular phospholipase A_2_ (*in*PLA_2_) activity and membrane mono- and polyunsaturated fatty acid (MUFA, PUFA) profile, within the UHR sample.

Based on previous findings in patients with established diagnosis of schizophrenia, where the majority of studies found attenuated niacin sensitivity [16,40], increased *in*PLA_2_ activity in blood plasma and post mortem brain tissue [41,42] and decreased PUFA levels [43–46], we postulated the following *a priori* hypotheses that: I) niacin sensitivity of our UHR sample will be attenuated, however to a lower extend than in FEP (in-between HCs and FEP), II) in the UHR sample niacin sensitivity will be positively correlated with membrane omega-6 fatty acid levels, in particular with arachidonic acid (AA), i.e. the lower the AA level, the weaker the niacin skin response, and III) niacin sensitivity will be negatively correlated with *in*PLA_2_ activity, i.e. the lower the niacin sensitivity, the higher the *in*PLA_2_ activity. IV) based on our recent findings of a dynamic interrelation between *in*PLA_2_ activity and PUFA levels between UHR non-transition (UHR-NT) and UHR transition (UHR-T) patients [47] we expected that niacin sensitivity will be more attenuated in the UHR-T group than in the UHR-NT group.

## Methods

### Participants

UHR subjects were recruited at the early psychosis detection unit of the Department of Child and Adolescent Psychiatry, Medical University of Vienna. All of them met UHR criteria for psychosis and participated in a randomised placebo-controlled trial (RCT) that investigates the influence of a 12 weeks omega-3 fatty acid supplementation (1.2g/d, including 700mg eicosapentaenoic acid (EPA) and 480mg docosahexaenoic acid (DHA)) on transition status, psychopathology and functioning within 12 month. In this study we present findings in baseline data of this RCT.

Among the inclusion and exclusion criteria, previously published in detail in the original publication of clinical findings [48], the following exclusion criteria were added for the niacin skin tests study: 1) recent use of non-steroidal anti-inflammatory drugs, 2) dermatological illness, such as psoriasis, eczema, atopical dermatitis, 3) medical illness that would affect normal vascular tone or response to niacin stimulus, such as inflammations, vasculitis, diabetes, or chronic hypertension, 4) non-Caucasian skin type and 5) low-quality photographs (insufficient sharpness, inappropriate exposure) that would prohibit “double-blind” ratings of the photographic documentation of the niacin skin reaction.

All consecutive referrals between April 2004 and May 2006 to the early detection unit of the University Hospital of Vienna were considered for inclusion. Two hundred fifty-six individuals were assessed for eligibility, 81 of whom met the inclusion criteria and consented to the original RCT (for details see [48]). A total of 65/81 UHR subjects in turn met the above mentioned requirements for the niacin skin test study. Detailed demographic information of this sample is provided in Table 1.

**Table 1:** Demographic and clinical characteristics of study participants.

	UHR-NT	UHR-T	FEP	HC
N	54	11	25	25
**Gender** (male/female)	18 (33.3%)	5 (45.5%)	17 (68.0%)	15 (60.0%)
**Age** (years) mean (±SD)	16.5 (±2.3)	16.4 (±1.3)	22.9 (±3.8)	32.9 (±7.4)
**Nicotine**				
No	27 (50.0%)	5 (45.5%)	8 (32.0%)	23 (92.0%)
≤ 10 cigarettes/d	13 (24.1%)	5 (45.5%)	10 (40.0%)	2 (8.0%)
> 10 cigarettes/d	15 (27.8%)	1 (9.1%)	7 (28%)	0
**Alcohol**				
No	0	0	0	8 (32.0%)
Less than weekly	31 (57.4%)	6 (54.5%)	13 (52.0%)	10 (40.0%)
1–6 drinks/week	16 (29.6%)	2 (18.2%)	8 (32.0%)	5 (20.0%)
Daily	8 (14.8%)	3 (27.3%)	4 (16.0%)	2 (8.0%)
**Marijuana**				
No	47 (87.0%)	9 (81.8%)	13 (52.0%)	25 (100%)
Less or equal 2 gr/week	4 (7.4%)	2 (18.2%)	12 (48.0%)	0
More than 2 gr/week	4 (7.4%)	0	0	0
**Drugs other than THC**	5 (9.3%)	2 (18.2%)	10 (40.0%)	0
**Psychiatric medication**				
Antidepressants	20 (37.0%)	5 (45.6%)	4 (16.0%)	0
Antipsychotic agents	0	0	25 (100%)	0
Benzodiazepine or sedative	9 (16.7%)	10 (90.0%)	8 (32%)	0
**Psychopathology**	** **			
PANSS positive subscale	14.3 (±3.3)	15.6 (±2.5)	20.12 (±7.41)	0
PANSS negative subscale	12.8 (±5.1)	18.5 (±6.9)	18.96 (±8.63)	0

NT = non-transition, T = transition, FEP = first-episode patient, HC = healthy control, all Caucasian

The institutional Research & Ethics Committee of the Medical University of Vienna, Austria, approved the study (EK-Nr.415/2002). All subjects (and for subjects under the age of 18 also their parents or legal guardians) gave written informed consent for the clinical interviews, the niacin sensitivity skin test, as well as for all performed biochemical analyses.

We also included a data set of 25 first-episode patients (FEP) in our analysis, all fulfilling DSM-IV criteria for schizophreniform psychosis or schizophrenia in order to perform a cross-sectional comparison between UHR and FEP subjects. These patients were investigated at the in- or outpatient clinic of Orygen Youth Health in Melbourne, Australia, using the same test protocol as at the Medical University Vienna, which was applied by the same researcher (St.S) and with the same equipment as in Vienna. Diagnosis, in- and exclusion criteria were confirmed by two independent experienced psychiatrists (St.S and G.B). Patients had a mean duration of illness of 14.5 months prior to performing the niacin skin test. All FEP patients were treated with atypical antipsychotic medication (olanzapine: n = 12, mean dose 9.6 mg/d, range 2-20mg/d; risperidone: n = 10, mean dose 3.1 mg/d, range 1–5 mg/d; quetiapine: n = 2, 600 mg and 800 mg/d; clozapine: n = 1, 300 mg/d).

We also included a niacin data set of 25 HC in our analysis, all recruited from staff of Orygen Youth Health to perform a cross-sectional comparison between UHR, FEP und HC. Current mental status, personal and family history of any mental disorder was assessed by the same two trained psychiatrist (St.S und G.B.) as in the FEP sample. Volunteers were not eligible as HC if they met any of the above mentioned exclusion criteria. FEP and HC were part of another niacin sensitivity study which compared two different methods to asses skin flush response (visual semi-quantitative vs optical reflection spectroscopy), and which included the investigation of medication effects [22]. The Research and Ethic Committee of the North Western Mental Health Network, Melbourne, Australia, approved this previous study. All participants gave written informed consent.

### Assessment of niacin sensitivity

Ward et al [17] were the first who applied a topical variant of the niacin skin test in psychiatry research. This test was further advanced to a test protocol that captures the information of intensity and time-course of skin redness and oedema using a descriptive seven point rating scale after stimulation with four niacin concentrations (0.0001M, 0.001M, 0.01M, and 0.1M), and at four assessment time points (5, 10, 15 and 20 minutes *post* niacin application). The descriptive rating scale defines 1 as *no skin reaction*, while 7 indicates *intense redness with visible oedema that starts to spread out or oedema bigger than the patch area*. The complete rating scale is provided as S1 File named Berger Niacin rating scale in the Supporting Information. Each rating was then entered in a matrix integrating all four concentrations and time points (16 scores in total), and enabled the calculation of an overall sum score as well as subscores for each concentration [22,49]. This descriptive assessment scale was evaluated using a method quantifying the skin reaction by optical reflection spectroscopy (see above [22]). The latter was not used in this study.

Using this test protocol, methylnicotinate (methyl pyridine-3-carboxylate, C_7_H_7_NO_2_, 99%, Sigma-ALDRICH Chemistry GmbH, Steinheim, Germany) was applied simultaneously in four dilutions (0.0001M, 0.001M, 0.01M, and 0.1M) of 50 μl each to four neighbouring places at the inner side of the forearm using commercial chambered plaster for epicutaneous testing. Methylnicotinate test solutions were freshly prepared for each test to prevent any influence of sunlight. After 60 sec of skin exposure to methylnicotinate the plaster was removed. Skin reaction was quantified before stimulation and in the areas of methylnicotinate exposure subsequently at 5-min intervals up to 20 minutes, starting 90 sec after removal of the methylnicotinate patches.

Statistical analysis was performed on the individual overall sum scores of these 16 ratings (possible min. /max. range between 16 and 112) in each participant, as well as on subscores for each concentration.

### Fatty acid analysis

Erythrocytes were separated from full blood to analyse fatty acid composition using the phosphatidylethanolamine (PE) fraction, as PE is the most common glycerophospholipid at the inner side of cerebral membranes. Using standard methods, values for saturated FA (SFA: 14:0, 16:0, 17:0, 18:0), monounsaturated fatty acids (MUFA including Trans-FA, TFAs: 18:1(ω-7)tr, 18:1(ω-9)tr, and omega-9 fatty acids: 18:1(ω-9), 20:1(ω-9), 22:1(ω-9), 24:1(ω-9)), and polyunsaturated fatty acids (PUFA including omega-6 fatty acids: 18:2(ω-6), 18:3(ω-6), 18:3(ω-6), 20:3(ω-6), 20:4(ω-6), 22:2(ω-6), 22:4(ω-6), and omega-3 fatty acids: 18:3(ω-3), 20:5(ω-3), 22:5(ω-3), 22:6(ω-3)) were obtained. More information on fatty acid analysis is provided in the publication of [47] and in the S2 File named Laboratory analyses of the Supporting Information.

### Intracellular Phospholipase A_2_ Activity (inPLA_2_)

*In*PLA_2_ activity in blood plasma was measured using a continuous kinetic fluorometric assay with the commercially available fluorescent substrate PED6 (Cat. No. D23739; In-Vitrogen, Carlsbad, California, USA). The assay strategy has been reported in the publication by Smesny et al, 2014 [47] and is also provided in detail in the S2 File named Laboratory analyses of the Supporting Information. Just briefly, PED6 incorporates a BODIPY^®^ FL dye-labelled sn-2 acyl chain and a dinitrophenyl quencher group. Cleavage of the dye-labelled acyl chain by *in*PLA_2_ eliminates the intra-molecular quenching effect of the dinitrophenyl group, resulting in a corresponding fluorescence increase, which is directly linked to *in*PLA_2_ activity. For calculating *in*PLA_2_ activities, the ascent of the fluorescence increase curve, the time interval after adding PED6 (slope/min), and calibration curves (based on commercial bee venom PLA_2_) were used. Resulting enzymatic activity was normalized to total protein concentration of the respective plasma sample. This yields specific activity (pmol/min)/mg protein). In order to measure *in*PLA_2_ requiring no or only a minimum amount of calcium, all measurements were conducted in a calcium depleted environment, established by adding ethylene glycol tetraacetic acid (EGTA) dissolution.

### Statistical analysis

#### Group comparison of niacin sensitivity data

All analyses were performed in baseline (pre-treatment) data of the original randomized controlled trial. As initial test we started with a multivariate analysis of variance (MANOVA) including sum values of each niacin concentration (0.0001M, 0.001M, 0.01M, and 0.1M) and the total niacin sum score as dependent variable, age (because being different between groups) as covariate and group (UHR non-transition [UHR-NT] n = 55, UHR transition [UHR-T] n = 11, first-episode patients [FEP] n = 25, healthy controls [HC] n = 25) as between-subject factor. Effects of single niacin concentrations were only considered if multivariate tests showed a significant effect of the factor group. To evaluate effect sizes we calculated partial eta^2^, considering 0.01 as small, 0.06 as moderate, and 0.14 as a large effect size.

We acknowledge the fact, that in all studies of our own group gender influenced the skin flush intensity. Therefore we performed all test in the entire sample as well as in males or females only. For the comparison of particular groups at particular niacin concentration we performed post-hoc tests. We only present Bonferroni corrected results.

#### Correlational analyses

Associations between niacin scores and age as well as psychopathology ratings were investigated by Pearson correlation analyses. In order to test for the assumed associations between niacin sensitivity and other biological parameters, Pearson correlation coefficients were calculated between niacin scores, sum values of fatty acid families (sum values of PUFA and MUFA as well as of omega-3, omega-6 and omega-9 FA) or values of single membrane fatty acids, and *in*PLA_2_ activity. The latter analysis was performed in UHR individuals only. Because of the explorative character of this analysis, we did at this state of knowledge not adjust for multiple tests.

## Results

### Sample characteristics

As expected, the mean age between the four groups was statistically significant different (F(3,115) = 98.73, p<0.001) (see Table 1). Testing for differences in age by non-parametric tests, significant differences could neither be found between the UHR-NT and UHR-T group, nor between the FEP and HC group.

The four groups did not differ in gender distribution (Chi-Square(3) = 6.826, p = 0.078). However, as an influence of gender on niacin sensitivity has been reported several times by our group, we present all results for the entire population, as well as for male or female subgroups.

### Niacin sensitivity in UHR-NT, UHR-T, FEP and HC and associations with psychopathology

Results of the multivariate and univariate tests of the initial MANOVA that was performed in the entire population as well as in the male and female subgroup are summarized in Table 2. Multivariate tests showed irrespective of gender a highly significant effect of the factor *group* but neither the factor *age* nor the *group by age interaction* was significant. However, the subgroup of females showed an influence of age on group effects at a trend level.

**Table 2:** Analysis of variance with niacin scores as dependent variable, age as covariable and group as between-subject factor.

		group			age		group by age	
		F	p	partial eta^2^	F	p	F	p
	**multivariate tests**							
**all**		6.904	**<0.001**	0.201	1.471	0.216	1.019	0.431
**males**		3.670	**<0.001**	0.231	1.163	0.341	0.575	0.860
**femals**		4.878	**<0.001**	0.255	1.141	0.348	*1*.*586*	*0*.*100*
	**univariate tests**							
**all**	0.0001M	10.176	**<0.001**	0.216	1.807	0.176	0.658	0.580
** **	0.001M	34.405	**<0.001**	0.482	0.404	0.526	0.703	0.552
** **	0.01M	26.487	**<0.001**	0.417	1.010	0.317	1.723	0.132
** **	0.1M	33.605	**<0.001**	0.477	0.743	0.390	1.361	0.259
** **	total sum value	33.476	**<0.001**	0.475	0.004	0.949	0.917	0.435
**males**	0.0001M	5.661	**0.002**	0.254	0.413	0.524	0.261	0.853
** **	0.001M	19.397	**<0.001**	0.538	1.675	0.202	0.727	0.541
** **	0.01M	9.040	**<0.001**	0.352	0.698	0.408	0.335	0.800
** **	0.1M	14.195	**<0.001**	0.460	1.669	0.203	0.574	0.635
** **	total sum value	17.309	**<0.001**	0.509	0.467	0.498	0.216	0.884
**females**	0.0001M	9.972	**<0.001**	0.340	0.057	0.812	0.347	0.791
** **	0.001M	29.827	**<0.001**	0.607	0.161	0.690	0.921	0.437
** **	0.01M	24.554	**<0.001**	0.559	2.303	0.135	2.911	**0.043**
** **	0.1M	26.052	**<0.001**	0.574	0.865	0.357	3.161	**0.032**
** **	total sum value	27.497	**<0.001**	0.587	0.315	0.577	1.185	0.324

Multivariate and univariate test results of MANOVA with niacin scores included as dependent variable, age as covariable and group as between-subject factor. Results are also presented for subgroups of males and femals. Bold numbers: results p < 0.05; italics: results at trend level p < 0.1.

Also results of the univariate tests indicate highly significant group effects in all niacin concentrations irrespective of gender. Corresponding to multivariate findings, group effects in females at the 0.01M and 0.1M niacin concentration are influenced by age.

To further explore the effect of age on niacin sensitivity, Pearson correlation analysis was performed in the entire population as well as in males and females [50] (total population: 0.0001M r = -0.337, p<0.001; 0.001M r = -0.427, p<0.001; 0.01M r = -0.306, p = 0.001; 0.1M r = -0.313, p = 0.001; total score r = -0.393, p<0.001; males: 0.0001M r = -0.364, p = 0.007; 0.001M r = -0.364, p = 0.007; 0.01M r = -0.206, p = 0.135; 0.1M r = -0.193, p = 0.162 total score r = -0.342, p = 0.011; females: 0.0001M r = -0.247, p = 0.053; 0.001M r = -0.451, p<0.001; 0.01M r = -0.350, p = 0.005; 0.1M r = -0.374, p = 0.003; total score r = -0.385, p = 0.002).

**Fig 1:**
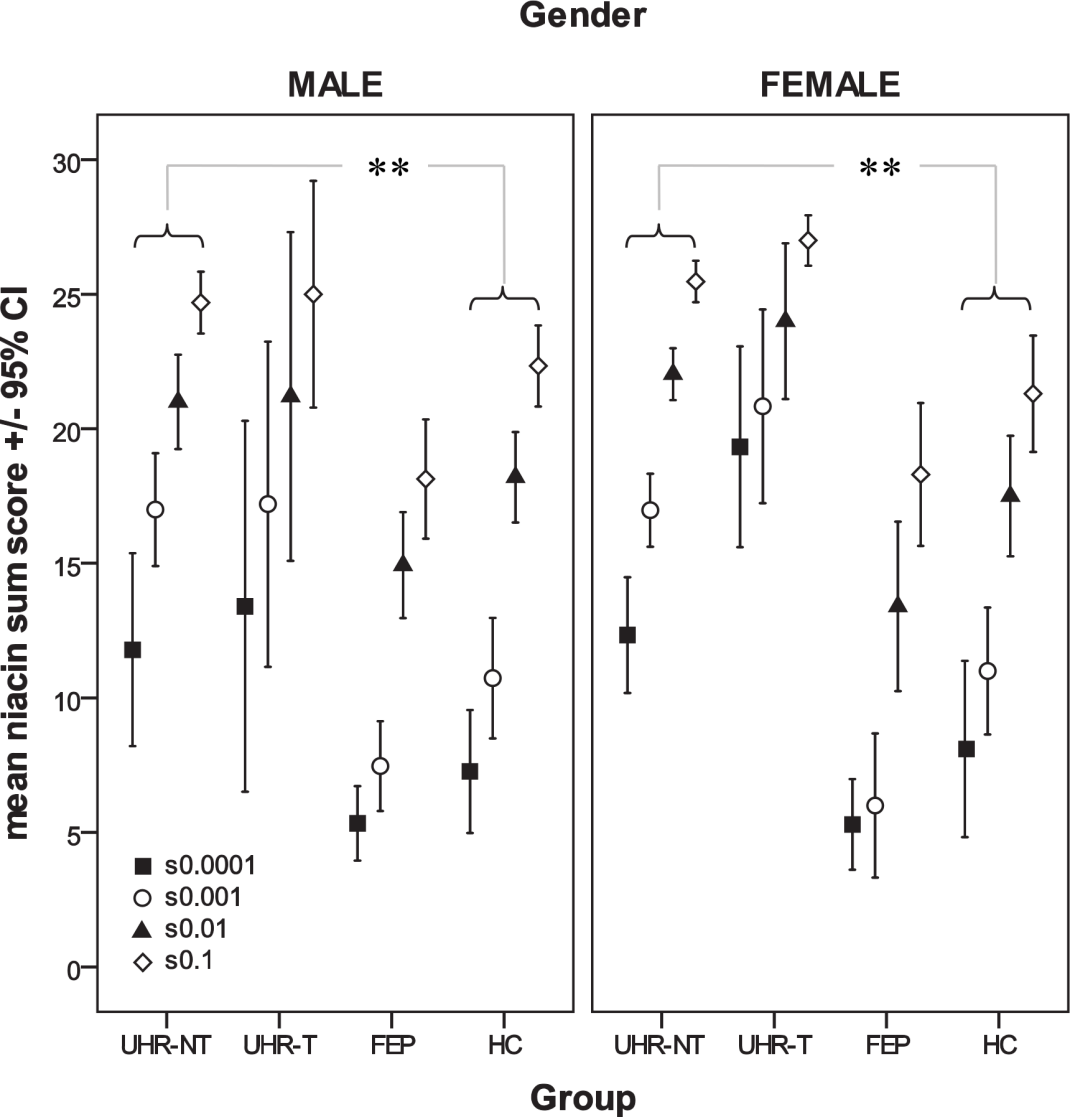
Illustration of group differences of niacin scores between non-transition (NT) and transition (T) UHR patients, first-episode psychosis patients (FEP) and healthy controls (HC). Due to obvious (although not significant) differences in gender distribution between groups and previously reported effects of gender on niacin skin sensitivity, results are presented separately for males and females. ** p<0.01.

Results of the *post-hoc* analyses corrected for multiple comparisons (Bonferroni) are summarized in Table 3. In general, niacin sensitivity of UHR individuals irrespective of the transition outcome was significantly higher than in HC (and in FEP). This finding seems to be even more pronounced in females. An illustration of the findings in total niacin scores of males and females is given in Fig 1. As a replication of previous findings, in all but the lowest concentration’s (0.0001M) sum scores, the flush response was significantly lower in FEP than in HC, equally apparent in males and females.

**Table 3:** Bonferroni corrected results of post-hoc comparison of single groups as revealed by MANOVA.

	all participants					males					females				
	mean	SD	mean	SD	p (corr.)	mean	SD	mean	SD	p (corr.)	mean	SD	mean	SD	p (corr.)
**niacin**	**UHR-NT** (n = 55)		**HC** (n = 25)		** **	**UHR-NT** (n = 19)		**HC** (n = 15)			**UHR-NT** (n = 36)		**HC** (n = 10)		
0.0001M	12.15	6.69	7.60	4.24	**0.004**	11.79	7.44	7.27	4.13	0.111	12.33	6.37	8.10	4.58	0.203
0.001M	16.98	4.09	10.84	3.69	**<0.001**	17.00	4.35	10.73	4.04	**<0.001**	16.97	4.01	11.00	3.30	**<0.001**
0.01M	21.67	3.14	17.92	3.03	**<0.001**	21.00	3.64	18.20	3.03	0.167	22.03	2.84	17.50	3.14	**0.001**
0.1M	25.20	2.32	21.92	2.83	**<0.001**	24.68	2.38	22.33	2.72	0.191	25.47	2.27	21.30	3.02	**<0.001**
Sum Score	76.00	14.10	58.28	9.91	**<0.001**	74.47	15.44	58.53	9.80	**0.003**	76.81	13.50	57.90	10.58	**0.001**
	**UHR-NT** (n = 55)		**UHR-T** (n = 11)		** **	**UHR-NT** (n = 19)		**UHR-T** (n = 5)			**UHR-NT** (n = 36)		**UHR-T** (n = 6)		
0.0001M	12.15	6.69	16.64	5.32	*0*.*080*	11.79	7.44	13.40	5.55	1.000	12.33	6.37	19.33	3.56	**0.030**
0.001M	16.98	4.09	19.18	4.35	0.533	17.00	4.35	17.20	4.87	1.000	16.97	4.01	20.83	3.43	0.153
0.01M	21.67	3.14	22.73	3.95	1.000	21.00	3.64	21.20	4.92	1.000	22.03	2.84	24.00	2.76	0.984
0.1M	25.20	2.32	26.09	2.47	1.000	24.68	2.38	25.00	3.39	1.000	25.47	2.27	27.00	0.89	1.000
Sum Score	76.00	14.10	84.64	14.58	0.240	74.47	15.44	76.80	17.08	1.000	76.81	13.50	91.17	8.89	*0*.*074*
	**UHR-NT** (n = 55)		**FEP** (n = 25)		** **	**UHR-NT** (n = 19)		**FEP** (n = 15)			**UHR-NT** (n = 36)		**FEP** (n = 10)		
0.0001M	12.15	6.69	5.32	2.39	**<0.001**	11.79	7.44	5.33	2.50	**0.006**	12.33	6.37	5.30	2.36	**0.004**
0.001M	16.98	4.09	6.88	3.33	**<0.001**	17.00	4.35	7.47	3.02	**<0.001**	16.97	4.01	6.00	3.74	**<0.001**
0.01M	21.67	3.14	14.32	3.90	**<0.001**	21.00	3.64	14.93	3.56	**<0.001**	22.03	2.84	13.40	4.40	**<0.001**
0.1M	25.20	2.32	18.20	3.81	**<0.001**	24.68	2.38	18.13	4.00	**<0.001**	25.47	2.27	18.30	3.71	**<0.001**
Sum Score	76.00	14.10	44.72	10.23	**<0.001**	74.47	15.44	45.87	8.56	**<0.001**	76.81	13.50	43.00	12.63	**<0.001**
	**FEP** (n = 25)		**HC** (n = 25)		** **	**FEP** (n = 15)		**HC** (n = 15)			**FEP** (n = 10)		**HC** (n = 10)		
0.0001M	5.32	2.39	7.60	4.24	0.831	5.33	2.50	7.27	4.13	1.000	5.30	2.36	8.10	4.58	1.000
0.001M	6.88	3.33	10.84	3.69	**0.003**	7.47	3.02	10.73	4.04	0.174	6.00	3.74	11.00	3.30	**0.029**
0.01M	14.32	3.90	17.92	3.03	**0.002**	14.93	3.56	18.20	3.03	*0*.*094*	13.40	4.40	17.50	3.14	**0.033**
0.1M	18.20	3.81	21.92	2.83	**<0.001**	18.13	4.00	22.33	2.72	**0.003**	18.30	3.71	21.30	3.02	*0*.*074*
Sum Score	44.72	10.23	58.28	9.91	**0.001**	45.87	8.56	58.53	9.80	**0.047**	43.00	12.63	57.90	10.58	*0*.*063*

Results are also presented for subgroups of males and females. Bold numbers: results p £ 0.05. Bold numbers: results p = 0.05; italics: results at trend level p = 0.1.

In UHR individuals there were no significant correlations between niacin flush scores and psychiatric symptom scores.

### Associations between niacin sensitivity and inPLA_2_ activity

**Fig 2:**
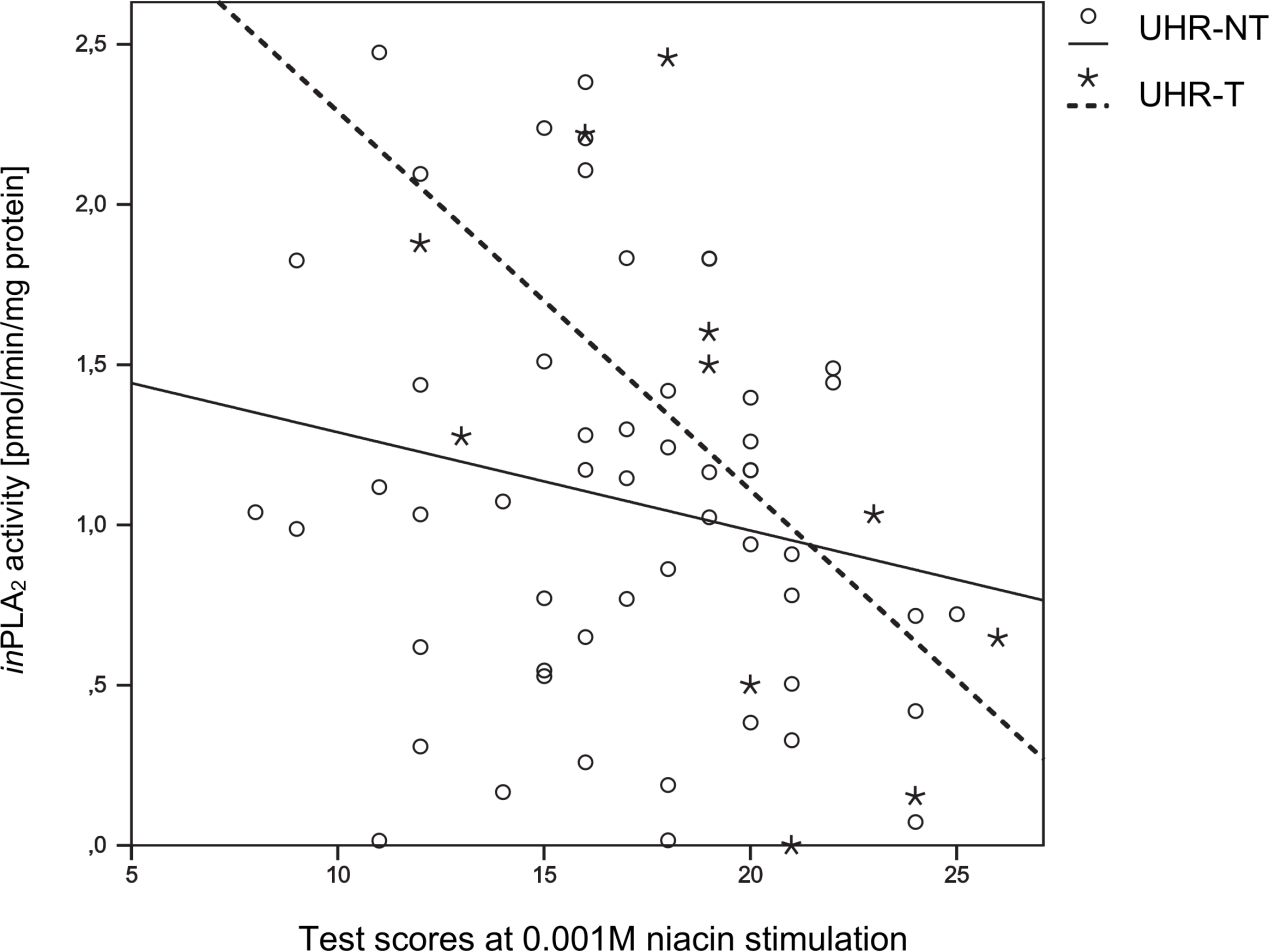
Results of correlation analysis between niacin sum scores at 0.001M stimulation and *in*PLA2 activity in UHR-NT- and UHR-T individuals.

The UHR group (n = 66) showed an inverse correlation between niacin sensitivity at 0.01M and 0.001M and *in*PLA_2_ activity (0.01M Pearson’s r = -0.294, p = 0.017; 0.001M r = -0.264, p = 0.034), i.e. decreased niacin sensitivity was associated with higher *in*PLA_2_ activity. As outlined in Fig 2, this correlation was even more pronounced if the analysis included only UHR-T individuals (n = 11; 0.001M r = -0.722, p = 0.028).

### Associations between niacin sensitivity and fatty acid levels

In UHR individuals (irrespective of transition outcome) sum values of niacin concentrations were not significantly correlated with sum values of omega-3 or omega-6 PUFA or omega-9 MUFA. However, niacin sensitivity correlated inversely with single omega-6 and omega-9 fatty acids (see Table 4). In all UHR individuals (n = 66) the omega-6 FAs gamma-linolenic acid (GLA, 18:3ω-6, precursor of arachidonic acid, AA) and docosadienoic acid (DSA, 22:2ω-6, metabolite of AA) were inversely correlated with niacin flushing after 0.01M or 0.1M stimulation. Of interest, GLA and DSA showed a significant positive correlation with *in*PLA_2_ activity (GLA r = 0.266, p = 0.023; DSA r = 0.253, p = 0.042).

**Table 4:** Correlation analysis between niacin sensitivity and membrane fatty acid levels between all UHR individuals.

	**Omega-6 PUFA**			
Niacin	18:3ω-6	22:2ω-6		
0.0001M	n.s.	n.s.		
0.001M	r = -0.263, p = 0.035	r = -0.260, p = 0.036		
0.01M	r = -0.328, p = 0.008	r = -0.358, p = 0.003		
0.1M	r = -0.340, p = 0.006	r = -0.346, p = 0.005		
Sum Score	n.s.	n.s.		
	**Omega-9 MUFA**			
	18:1ω-9trans	20:1ω-9	20:3ω-9	22:1ω-9
0.0001M	r = -0.247, p = 0.047	n.s.	r = -0.250, p = 0.045	n.s.
0.001M	r = -0.285, p = 0.021	n.s.	r = -0.285, p = 0.021	n.s.
0.01M	n.s.	r = -0.337, p = 0.006	r = -0.299, p = 0.015	r = -0.364, p = 0.003
0.1M	n.s.	r = -0.329, p = 0.007	r = -0.333, p = 0.007	r = -0.356, p = 0.004
Sum Score	r = -0.248, p = 0.046	n.s.	n.s.	n.s.

gamma-linolenic acid (18:3ω-6), docosadienoic acid (22:2ω-6), oleic acid (18:1ω-9trans), eicosenoic acid (20:1ω-9), mead acid (20:3ω-9), erucic acid (22:1ω-9).

In omega-9 MUFA, three single FA (see Table 4) were inversely correlated with skin flush response after 0.01M and 0.1M stimulation. Also oleic acid and eicosenoic acid were positively correlated with *in*PLA_2_ activity (oleic acid r = 0.242, p = 0.052; eicosenoic acid r = 0.261, p = 0.036).

## Discussion

In summary, there were three main findings. First, in contrast to the results in established schizophrenia and also to our initially formulated hypotheses, niacin sensitivity is not attenuated, but significantly increased in our UHR compared to HC and FEP groups (see Fig 1). Second, also contradictory to our hypotheses, within our UHR sample an inverse correlations between niacin sensitivity and levels of single omega-6 and 9 fatty acids are present. Finally, in line with our hypothesis lower niacin sensitivity is associated with a higher *in*PLA_2_ activity.

We believe that the finding of increased niacin sensitivity in conjunction with decreased omega-6 and omega-9 FA in UHR individuals may be a signifier of a range of interrelated pathomechanisms (e.g. oxidative stress) resulting in an increased demand of bioactive lipids or an adaptive function associated with the emergence of psychosis. Having not investigated cerebral processes in this study, we speculate i) that an increased release/demand or an adaptive down regulation of AA and (to a smaller extend) also DGLA could relate to an unspecific “proinflammatory” process due to the excessive synaptic elimination and the associated pruning {Feinberg, 1982 #1895;Feinberg, 1990 #1894;Keshavan, 1994 #1232;Farooqui, 2007 #9895;[51] or ii) to excessive production of reactive oxygen species in the context of a weakened antioxidative defense mechanisms, e.g. through a dysfunctional glutathione cycle [52–57].

Indeed, outside the central nervous system, AA and their bioactive lipids play a crucial role for inflammatory processes. However, in the brain they are key players for the regulation of neurodevelopment, long-term potentiation, synaptic plasticity and excitatory neurotransmission [58–60]. Having in mind the preliminary character of our findings, the inverse correlations between niacin sensitivity (that occurs as a result of a prostaglandin mediated pathway) and omega-6 or -9 FA could be interpreted as follows: Increased niacin sensitivity in UHR individuals could signify an increased demand for omega-6 FA (in particular for AA and its metabolites) and in the due course also for omega-9 FA resources, since monounsaturated omega-9 FA serve as compensatory replacements once omega-6 FA are exhausted [47]. Although providing some explanation for the increased niacin sensitivity in UHR individuals, this pathology seems not yet very pronounced in our UHR sample, as inverse correlations were observed only in single FA, but not in sum values of omega-6 FA, PUFA or MUFA. Thus, while bioactive lipids and fatty acids seem to be still in balance in our UHR population due to effects of compensation, the attenuated niacin sensitivity observed in FEP may then be the result of manifest exhaustion of omega-6 FA resources and the incapacity to compensate the PUFA deficiency. This interpretation is also supported by our observation of an inverse correlation between niacin sensitivity and *in*PLA_2_ activity, and by the positive correlations between single omega-6 and omega-9 FA and *in*PLA_2_ activity respectively, as increasing *in*PLA_2_ activity is counted as marker of up-regulated membrane lipid repair/remodeling processes [61] and was repeatedly found in patients suffering schizophrenia [42].

Another line of research suggests that regulatory deficits in immune function are present in schizophrenia and related disorders [62]. As mentioned earlier, niacin sensitivity is regulated by prostaglandins (PGD_2_, PGE_1_), mainly metabolites of AA that is released by *in*PLA_2_ and further metabolized mainly by COX-1 and COX-2. Considering this pathway, it is also possible that increased niacin sensitivity may signify altered immunomodulatory activity in UHR individuals, i.e. prior to the onset of psychosis. Such an idea may also provide explanation for our findings, and may support the idea that progression to acute psychosis is accompanied by increased need of AA and its derivatives (such as eicosanoids) [63,64].

In contradiction to our hypothesis (IV), niacin sensitivity did not significantly differ between UHR-transition (T) and -non-transition (NT) individuals. We also treat the difference at trend level found at the lowest niacin concentration (0.0001M) with caution, as even the solid finding of decreased niacin sensitivity in FEP got lost at this lowest niacin concentration. Thus, the lowest niacin concentration seems to have in general the lowest sensitivity to disclose group effects.

A range of studies of our group investigating the underlying neurobiology of the emergence of psychosis are in line with the proposed interpretations: First, using ^1^H-MR spectroscopy imaging [65] omega-3 fatty acids induced the in vivo brain glutathione levels by 30%, a strong intracellular antioxidants often induced during inflammation [55,66]. Furthermore T2 relaxation time [67], a very sensitive albeit unspecific marker of active potentially proinflammatory processes, was increased in UHR individuals [65,67]. Second, nervonic acid, a monounsaturated omega-9 FA crucially involved in myelination and a signifier of an exhaustion of proinflammatory metabolites, was decreased in UHR individuals, in particular in those suffering transition to acute psychosis within one year [68]. Finally, early prenatal and/or perinatal exposure to various environmental insults, including maternal exposure to stress, infections, nutritional deficiencies as well as obstetric complications were found associated with increased demands of PUFAs [51].

Our study has several limitations: Age and gender may be potential confounders, as niacin sensitivity is generally more pronounced in females and attenuates gradually with age as shown previously by our group [50]. This was also found in this study. In particular in females, age significantly influenced group effects. Another potential confounder could be substance abuse that was overexpressed in our UHR group. Our group was able to demonstrate an inverse association between niacin sensitivity and cannabis abuse (niacin sensitivity decreased in consuming healthy controls) [69]. Furthermore, pharmacological treatment could have affected niacin sensitivity. The use of antidepressants and benzodiazepines was higher in the treated UHR group and therefore could have affected PUFA/oxylipin metabolism and the HPA-axis. As effects of these agents on niacin sensitivity have not been shown or systematically investigated as yet, a potential contribution to our results cannot be excluded at this stage of knowledge.

Furthermore, the UHR population is rather heterogeneous. Current evidence suggests that niacin sensitivity is decreased in schizophrenia, may be also decreased to a lesser extend in unipolar depressive disorder, autism, dyslexia and hyperactivity disorder, but seems to be not changed or even increased in manic episodes of bipolar affective disorders [16,70]. In depression, this pathology is linked to hypothalamic-pituitary-adrenal (HPA) axis dysfunction, showing ongoing (nocturnal) ACTH and cortisol secretion (as caused by HPA axis hyperactivity) to disturb the balance between omega-6 and omega-3 PUFA [71,72], which may in turn causes down-stream effects on prostaglandin-mediated pathways. In the context of this study, the findings that niacin flush response was positively correlated with severity of melancholic features, anxiety, feelings of guilt and somatic concerns is interesting [70,73], depression is quite common in UHR [74–76]. Therefore, niacin sensitivity in UHR individuals may be influenced by the composition of the UHR population and the spectrum/severity of symptomatology. In our particular UHR cohort from Vienna, most converters developed schizophrenia (see consort diagram of the clinical trial publication by [48]), however this may not be the case in other UHR populations. We were not able to show associations between niacin sensitivity and psychopathology, presumably due to the limited range in symptom severity in this UHR sample. For future niacin test studies, it could be important to stratify the study population according to UHR subgroups, spectrum/severity of symptoms as well as according to the long-term diagnosis.

In summary, in this study UHR subjects showed increased while FEP showed decreased sensitivity to niacin [23]. The presented (weak) associations with omega-6- and omega-9 FA metabolism and with PLA_2_ activity suggest niacin sensitivity may indicate that the UHR state may be associated with a “pro-inflammatory state”. Future research should address the question if the UHR state is associated with an incipient neurovulnerability that is associated with bioactive lipid abnormalities and an activation of neuro-inflammatory processes. Potential implications for neuroprotective treatments should be addressed. [67,77].

**Highlights**

1. Niacin sensitivity, an established marker of disturbed prostaglandin metabolism, is increased in individuals at ultra-high risk for an acute psychosis.
2. The increased niacin sensitivity in the ultra-high risk phase to acute psychosis seems to be a reflection of increased omega-6- and omega-9 fatty acid demand and of an up-regulated membrane fatty acid turnover.
3. The increased niacin sensitivity in individuals at ultra-high risk for psychosis as compared to patients with established schizophrenia may be a signifier of a “pro-inflammatory state” associated with the emergence of psychosis.
4. In case of replication, niacin sensitivity could potentially be useful to identify UHR individuals who would benefit from neuroprotective treatment options targeting lipid oxidation or pathways of inflammation.

## Supporting Information

S1 File Berger Niacin rating scale. (DOC)

S2 File Laboratory analyses. (DOCX)

S3 File SPSS Database Berger et al PLosOne 2015. (SAV)
