# Supplementary material for: Niacin Skin Sensitivity Is Increased in Adolescents at Ultra-High Risk for Psychosis
Source: PLoS One. 2016 Feb 19;11(2):e0148429. doi: 10.1371/journal.pone.0148429 (PMC4764507; doi:10.1371/journal.pone.0148429)
Supplement: S1 File — (DOC) [file pone.0148429.s001.doc]

S1 Berger Niacin Rating Scale

1. no skin reaction at all
2. red spots within niacin patch area, confluence of red spots less than 50% of total area, patch area not sharp, and no oedema
3. slight redness within niacin patch area; whole patch area can now be recognized, no oedema, confluence of red spots more than 50% of patch area
4. moderate redness within niacin patch area; patch area is now sharp and whole patch area has a homogeneous erythema, and/or cut-off point for beginning oedema (one can feel a slight elevation, but not necessarily see it)
5. redness starts to spread out (‘corona’), and/or oedema now visible, slight contrast between patch area and surrounding, but patch area has still the same color as ‘corona’, ‘patch area border’ is still sharp
6. redness in patch area has turned from red to orange/yellow, and/or redness around the oedema clearly separates patch area from ‘corona’, and/or clearly visible oedema, encompassing whole patch area, ‘patch area border’ is becoming diffuse
7. marked redness that clearly spreads out (patch area nearly not recognizable anymore), and clearly visible oedema that starts to spread out (oedema bigger than original patch area)
