# Supplementary material for: Niacin Skin Sensitivity Is Increased in Adolescents at Ultra-High Risk for Psychosis
Source: PLoS One. 2016 Feb 19;11(2):e0148429. doi: 10.1371/journal.pone.0148429 (PMC4764507; doi:10.1371/journal.pone.0148429)
Supplement: S2 File — (DOCX) [file pone.0148429.s002.docx]

S2 Laboratory analyses

*Acquiring/storage of erythrocyte membrane samples*

Phospholipid fractions for the analysis of the diverse membrane fatty acids were extracted from erythrocyte membranes, which were prepared as follows: Blood was taken right after the niacin patch test from an antecubital vein of the non-tested arm and was then centrifuged (3,000 rpm, 20 min). After separation of plasma for measurement of *in*PLA_2_ activity, erythrocytes were washed 3 times (each time 10 min, 3,000 rpm) in 4 ml isotonic phosphate buffer (isotonic buffer: 1.42 g Na_2_HPO_4_; 7.27 g NaCl, and 0.19 g Na-EDTA dissolved in ~950 ml distilled H_2_O; pH adjusted to 7.4 using 1M HCl, volume corrected to 1,000 ml with distilled H_2_O).

To avoid oxidation processes, hemoglobin was washed out stepwise after hemolysis using Tris buffer (11 mM, pH 7.6) and an extracting agent (methanol:chloroform (1:2) + 50 mg/l BHT, both obtained from Merck KGaA Darmstadt; butylated hydroxytoluene; min 99.0%; Sigma^®^). As an internal standard, 200 μl Tocol was added (concentration 10 μg/ml). Using the method of Dodge et al. 1967 ([Dodge *et al* 1967](#_ENREF_1); [Dodge and Phillips 1967](#_ENREF_2)), erythrocyte membranes were isolated stepwise during three rounds of hemolysis (20 ml Tris buffer, 5 min) and centrifugation (13,000 rpm, 10 min, 4°C, Ultraspeedcentrifuge Sorval^®^ Ultra Pro 80), each time after removal of the supernatant. Erythrocyte membrane samples were stored at -80°C after N_2_ gassing.

*Analysis of fatty acids (FAs)*

Fatty acid-containing erythrocyte membrane extracts were evaporated, and phospholipid fractions (sphingolipids, phosphatidylcholine (PC), phosphatidylserine (PS), phosphatidylinositole (PI), phosphatidylethanolamine (PE)) were dissolved using thin-layer chromatograpohy (50 ml chloroform, 37.5 ml methanol, 3.5 ml glacial acetic acid, and 2 ml destilled water). Phospholipid fractions were now saponified and esterified.

The PC fraction of membrane phospholipids in the CNS is situated primarily on the outer side of membranes, and includes mainly saturated FAs. In contrast, the PE fraction is situated largely on the inner side of membranes, with its main FAs being palmitic acid (16:0), stearic acid (18:0), oleic acid (18:1 ω-9), and arachidonic acid (20:4 ω-6). The smallest percentage of membrane phospholipids falls in the PS fraction, which is also situated on the inner side of the membrane, and includes stearic acid (18:0), and arachidonic acid (20:4 ω-6).

The presumed pathology investigated in this study presumably affects mainly the cytosolic (inner) side of membranes. As PE is the most common phospholipid on the inner side of membranes in the brain, we only included the FA levels (in mol% of total fatty acid levels) of this PE fraction in our statistical analysis.

FAs were dissolved from erythrocyte membranes using the extracting agent (methanol:chloroform (1:2) + 50 mg/l BHT), followed by separation of the lipid phase from the water-soluble phase using a separating funnel (method according to Folch et al. 1957 ([Folch *et al* 1951](#_ENREF_3); [Folch *et al* 1957](#_ENREF_4); [Folch *et al* 1958](#_ENREF_5))).

FA methyl esters were then resolved in hexane and injected into the gas-phase chromatograph (Clarus 500; PE AutoSystem). The following FA were quantified using commercial standards: saturated FA (SFA: 14:0, 16:0, 17:0, 18:0), monounsaturated fatty acids (MUFA: 18:1ω-9, 20:1ω-9, 20:3ω-9, 22:1ω-9, 24:1ω-9), trans fatty acids (TFA: 18:1ω-7tr, 18:1n-9tr), and PUFA (18:2ω-6, 18:3ω-6, 20:3ω-6, 20:4ω-6, 22:2ω-6, 22:4ω-6, 18:3ω-3, 20:5ω-3, 22:5ω-3, 22:6ω-3).

*Analysis of inPLA_2_-activity*

Plasma *in*PLA*_2_* activity was measured using a continuous kinetic fluorometric assay. We used the commercially available fluorescent substrate PED6 (Cat. No. D23739; In-Vitrogen, Carlsbad, California, USA; N-((6-(2,4-dinitrophenyl)amino) hexanoyl)-2-(4,4-difluoro-5,7-dimethyl-4-bora-3a,4a-diaza-s-indacene-3-pentanoyl)-1-hexa-de-canoyl-sn-glycero-3-phosphoethanolamine). PED6 incorporates a BODIPY^®^ FL dye-labeled sn-2 acyl chain and a dinitrophenyl quencher group. Cleavage of the dye-labeled acyl chain by *in*PLA_2_ eliminates the intramolecular quenching effect of the dinitrophenyl group, resulting in a corresponding increase in fluorescence ([Hendrickson *et al* 1999](#_ENREF_6)). Thus, the measured fluorescence intensity kinetics are directly linked to *in*PLA_2_ activity. The fluorescent reaction product has its maximum absorption at 505 nm, and its maximum emission at 515 nm. Fluorescence was measured using a microplate reader (FLUOstar Omega, BMG LABTECH GmbH, Offenburg, Germany), equipped to pipette and to dispense reagents automatically. We used a filter combination of Ex 485 nm/Em 520 nm.

In order to measure intracellular calcium-independent PLA_2_ (*in*PLA_2_) exclusively, all measurements were conducted in a calcium-depleted environment, established by adding ethylene glycol tetra acetic acid (EGTA) to the reactions. For calibration we used a standard dilution series of bee venom PLA_2_ (SIGMA 29279-1MG). The wells of a 96-well microplate were filled with HEPES buffer, then 5 µl of plasma or the respective standard solution were added. After recording baseline values, 5 µl of PED6 solution (dissolved in dimethyl sulfoxide to obtain a 200 mM stock solution) were added via the reagent dispenser. The total measurement time was 70 seconds per well. For calculating *in*PLA_2_ activities, the ascent of the curve and time interval after adding PED6 (slope/min) and calibration curves were used. The resulting enzyme activity was normalized to the total protein concentration of the respective plasma sample. This yields specific activity in (pmol/min)/mg protein.

**References**

Dodge JT, Cohen G, Kayden HJ, Phillips GB (1967) Peroxidative hemolysis of red blood cells from patients with abetalipoproteinemia (acanthocytosis). *J Clin Invest* 46:357-68

Dodge JT, Phillips GB (1967) Composition of phospholipids and of phospholipid fatty acids and aldehydes in human red cells. *J Lipid Res* 8:667-75

Folch J, Ascoli I, Lees M, Meath JA, Le BN (1951) Preparation of lipide extracts from brain tissue. *J Biol Chem* 191:833-41

Folch J, Lees M, Sloane Stanley GH (1957) A simple method for the isolation and purification of total lipides from animal tissues. *J Biol Chem* 226:497-509

Folch J, Lees M, Carr S (1958) Studies of the chemical composition of the nervous system. *Exp Cell Res* 14:58-71

Hendrickson HS, Hendrickson EK, Johnson ID, Farber SA (1999) Intramolecularly quenched BODIPY-labeled phospholipid analogs in phospholipase A(2) and platelet-activating factor acetylhydrolase assays and in vivo fluorescence imaging. *Anal Biochem* 276:27-35
